# Household food practices and implications for food security and child feeding in Timor-Leste

**DOI:** 10.3389/fpubh.2026.1852471

**Published:** 2026-09-07

**Authors:** Md Abu Sayeed, Lesy Careca Atok, Salvador Amaral, Arcenio Bano do Nascimento, Francisca Soares Alfonso, Deolinda Gusmão Soares, Angus McLure, Aparna Lal, Haribondhu Sarma, Kathryn Bright, Adriano Barbosa, Marcelo Amaral Mali, Shawn Ting, Md Rezanur Rahaman, Samantha Colquhoun

**Affiliations:** 1 National Centre for Epidemiology and Population Health (NCEPH), The Australian National University, Canberra, ACT, Australia; 2 Menzies School of Health Research, Dili, Timor-Leste; 3 Instituto Nacional de Saúde Pública de Timor-Leste (INSP-TL), Dili, Timor-Leste; 4 Global and Tropical Health Division, Menzies School of Health Research, Charles Darwin University, Dili, Timor-Leste

**Keywords:** caregiving, child nutrition, food security, gender, household food practices, qualitative research, social determinants, Timor-Leste

## Abstract

**Introduction:**

Household food security and food preparation practices are shaped by social roles, caregiving responsibilities, and economic conditions. We examined everyday household food practices in Timor-Leste within systems of caregiving, authority, and resource constraints, and their implications for household food security and child feeding.

**Methods:**

We conducted an exploratory qualitative study from August to October 2025 using semi-structured, in-depth interviews with mothers who had caregiving responsibilities for children in their households across urban Dili and the rural municipalities of Ainaro and Ermera. Data were analysed using a hybrid deductive–inductive thematic approach. A total of 34 mothers participated in the study.

**Results:**

Food practices were organised through women’s caregiving roles, household decision-making, and economic constraints. Women coordinated everyday food work and maintained food adequacy even under financial strain. Food allocation followed a hierarchy but remained flexible, with children prioritised during periods of scarcity. Neighbourhood sharing functioned as an informal redistribution system shaped by reciprocity and social obligation. Ceremonial obligations reinforced social cohesion but introduced financial pressure. Meal frequency and food choices were shaped by income stability, while caregiving authority determined the prioritisation of limited resources. Food knowledge was transmitted intergenerationally, and diets combined traditional staples with selected market foods.

**Discussion:**

Household food practices in Timor-Leste are shaped by caregiving roles, social relationships, and economic constraints, with implications for household food security and child feeding. These findings identify caregiving, food allocation, and adaptive responses to resource constraints as household processes potentially relevant to child nutrition vulnerability. Interventions that recognise women’s central roles in everyday food management, stabilise income, and build on reciprocal systems may support household food security and child feeding.

## 1 Introduction

Food is central to family and community life in Timor-Leste, where it not only satisfies hunger but also reflects hierarchy, strengthens bonds, and reinforces cultural values (1). In Timorese society, sharing food represents respect, care, and belonging. The act of serving a meal is perceived as an expression of hospitality, whereas refusing food is often interpreted as an act of disrespect or a breach of social norms (1). Cooking, serving, and consuming meals together represent values of unity and interdependence among Timorese families (2). Mealtime in Timor-Leste therefore extends beyond nutrition and structures hierarchy, belonging, reciprocity, and collective identity across communities (3, 4).

Meals often include traditional dishes such as corn, rice, porridge, grilled fish, and steamed banana-leaf parcels, which symbolise family bonds and ritual meaning (3). Additionally, food becomes a medium of spiritual connection in Timorese communities, often established through offerings to ancestral spirits during weddings and harvest blessing ceremonies that reinforce respect for ancestry and cultural heritage (4). Despite this rich food culture, nearly 46% of children under five are stunted, illustrating the coexistence of strong cultural food traditions and persistent nutritional vulnerability (5). Therefore, understanding these everyday practices may inform public health interventions seeking to support household food security and child feeding in Timor-Leste.

Timor-Leste is a country of mountainous terrain and erratic rainfall, which limits agricultural productivity and reduces food diversity, especially among households that primarily depend on subsistence farming (5). While rural families grow maize, cassava, taro, and beans for consumption, they often purchase rice from the market, linking household nutrition to cash flow and market access (6). Moreover, women are central to food dynamics, acting as both caretakers and custodians of culinary knowledge, responsible for meal planning, cooking, portion distribution, and the management of leftovers (3). Men are often described in the literature as influencing decisions around income use or livestock slaughter, creating a gendered division of labour rooted in Timorese social structures that have evolved from pre-colonial to contemporary times (4). More broadly, food allocation may reflect expectations of respect and hierarchy, with serving order expressing culturally recognised social relationships (7). Women’s influence over everyday food practices runs alongside men’s control over income and assets, creating a system of shared responsibility within unequal power relations (3, 5).

As the pressures of modernisation and globalisation infiltrate traditional food systems, longstanding practices are undergoing gradual transformation. Urbanisation has increased exposure to global markets and shifted dietary preferences, particularly in cities like Dili, where convenience foods and imported items are increasingly replacing traditional cuisines (3, 4). These transformations not only alter dietary quality but also reshape household food practices and family roles, as shared meal preparation becomes less common (4). Generational change may also shape food practices, as younger family members adopt different food preferences and traditional food practices become more difficult to maintain across generations (8). This tension illustrates how households negotiate between continuity and changing food preferences. Moreover, these differences show how food remains a site where cultural identity and social change intersect (3, 4).

Nutrition in Timor-Leste cannot be explained by income or dietary indicators alone; it also depends on the organisation, distribution, and management of food within households. Household food practices are increasingly understood as socially organised processes through which caregiving responsibilities, household authority, reciprocity, and resource allocation are negotiated, rather than simply as individual dietary behaviours (9–11). This relational perspective is consistent with moral economy theory, which draws attention to the social norms, responsibilities, and expectations of fairness that influence economic and domestic practices (12). In the context of household food practices, this perspective provides a useful lens for examining how food provisioning and allocation are negotiated through caregiving responsibilities, reciprocity, obligation, and perceptions of need under conditions of resource constraint. Moreover, these everyday practices have implications for food security and child feeding, particularly in settings constrained by social and ecological pressures. Understanding food security therefore requires attention to food allocation and management within households, rather than relying solely on aggregate indicators. Despite growing attention to food security and child nutrition in Timor-Leste, few studies examine everyday household food practices under conditions of economic constraint and social obligation, and limited evidence addresses the role of caregiving responsibilities, household decision-making, and resource limitations in shaping these practices.

Therefore, this study examines everyday household food practices in three municipalities in Timor-Leste, focusing on how decisions about food allocation, prioritisation, and caregiving are organised within households under conditions of economic constraint. The study aims to understand these practices and their implications for household food security and child feeding in low-resource settings.

## 2 Materials and methods

### 2.1 Study design and participants

**Figure 1:**
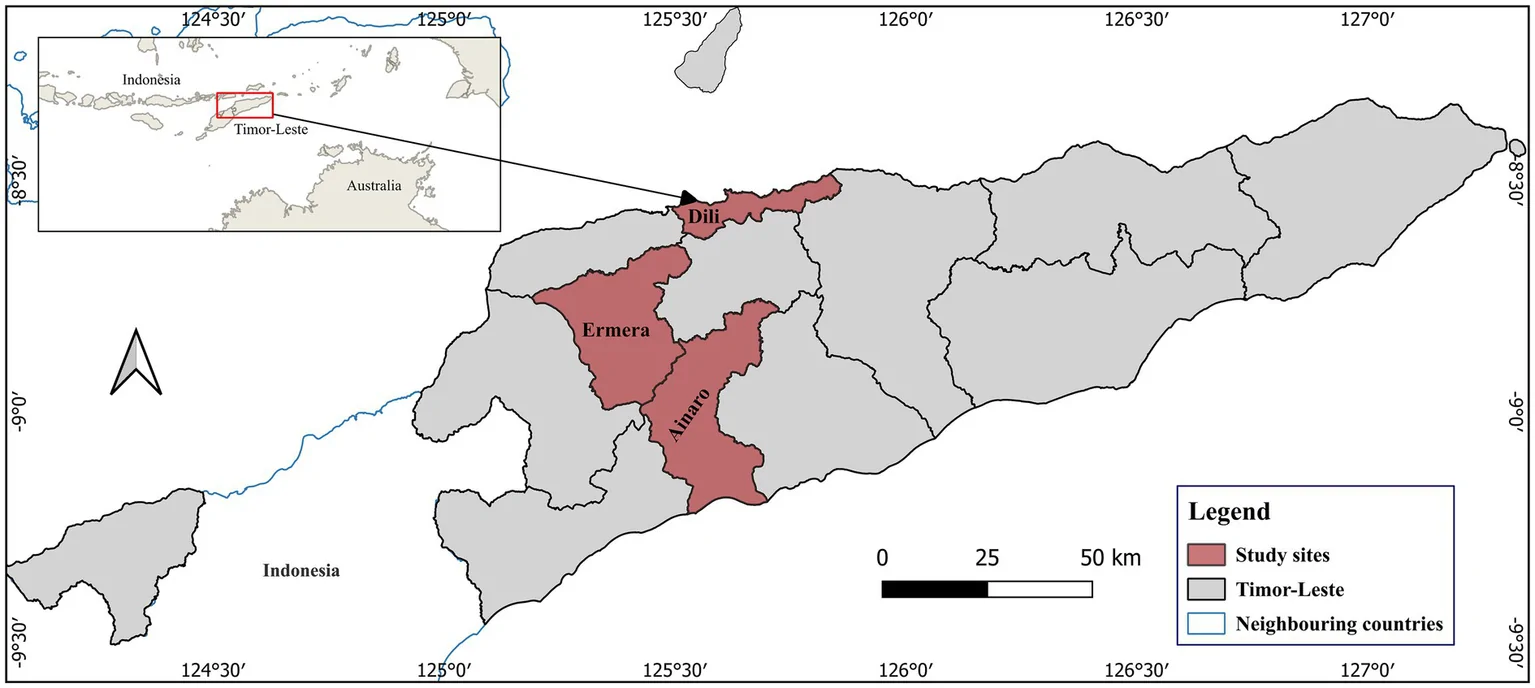
Geographic location of the three study municipalities in Timor-Leste (Dili, Ainaro, and Ermera). The map was created using a shapefile from the freely accessible GADM database (GADM; www.gadm.org) (17).

This study used an exploratory qualitative design informed by an interpretivist epistemology that prioritises understanding how social meaning, caregiving responsibility, and authority are constructed and negotiated within everyday household practices in Timor-Leste. This approach enabled exploration of participants’ experiences and the meanings they attributed to household food practices within their social and cultural contexts, rather than testing predetermined relationships. The study formed part of a broader qualitative research project titled “Exploring the influence of cultural norms, practices, and community factors on food choices, preparation, and food storage in the Timorese community” which is an embedded study under the Bacteria, Enteropathy, and Nutrition (BEN) research programme (13). We conducted semi-structured in-depth interviews at the individual level in Dili, Ainaro and Ermera municipalities in Timor-Leste (Figure 1). The three municipalities were purposively selected to capture contrasting urban and rural settings and variation in geographic, infrastructure, and livelihood contexts. Dili, the national capital and principal urban centre, represented the urban setting, whereas Ainaro and Ermera provided predominantly rural settings characterised by mountainous terrain and differing patterns of road accessibility and agricultural livelihoods (14, 15). More broadly, rural livelihoods in Timor-Leste remain strongly linked to agriculture, while limited market access and infrastructure are recognised challenges within the national food system (16). However, the urban–rural classification was used descriptively to characterise the recruitment settings rather than as a basis for formal comparative analysis.

Participants were mothers with caregiving responsibilities for children in their households and were purposively selected because of their direct and routine involvement in everyday food preparation, feeding, and household food management. The study therefore prioritised participants with first-hand experience of these practices rather than seeking maximum variation by gender. Participants were recruited by the research team, with the assistance of community leaders, mother support networks, and health workers.

### 2.2 Data collection

Interviews were conducted in Tetum between August and October 2025 using a flexible semi-structured interview guide that ensured consistency while allowing participants to emphasise issues most relevant to their household experiences. The principal investigators and the BEN Timorese research team developed the interview guide with reference to relevant literature on household food practices, food acquisition and consumption, intra-household food allocation, and gendered decision-making in Timor-Leste (1, 18, 19), together with expert input from researchers working on household food practices, caregiving, and gendered domestic labour. Questions explored food acquisition, preparation, storage, allocation, feeding practices, water use, and other aspects of household food practices. The guide was developed for the broader qualitative research project and covered multiple domains of household food practices. The present analysis focuses on household food allocation, caregiving responsibilities, and decision-making under economic constraint.

The interview guide was pre-tested by research nurses in Timor-Leste to ensure clarity and cultural appropriateness. It was further refined during the first eight interviews conducted in Dili. These interviews informed minor adjustments to wording and probes without altering the core research inquiry and were therefore included in the final analysis. The final interview guide is provided in Supplementary File 1.

Interviews were conducted by trained Timorese members of the local BEN research team who were familiar with the local language and cultural context. Interviews took place in participants’ homes or other locations chosen by participants, lasted approximately 45–90 min, and were audio-recorded with informed consent. Reflexivity was considered throughout data collection and analysis, with attention to how researchers’ professional, cultural, and disciplinary positions and interactions with participants could shape data generation and interpretation. All interviews were transcribed verbatim in Tetum and translated into English. Bilingual members of the research team reviewed translations to ensure conceptual and cultural accuracy. A subset of transcripts was independently back-translated into Tetum to verify translation accuracy. Any discrepancies were discussed and resolved through team consensus to preserve meaning.

Recruitment continued until thematic patterns became repetitive and no substantially new concepts emerged. We assessed thematic sufficiency through team discussions and review of coded transcripts to determine whether additional interviews contributed meaningful insights to emerging themes.

### 2.3 Data analysis

Data were analysed using a hybrid thematic approach combining deductive structuring with inductive refinement. This approach was considered appropriate because the study was guided by predefined areas of interest relating to household food practices while also seeking to capture participants’ perspectives and unexpected insights that emerged from the data. We first organised the analysis around broad *a priori* coding domains derived from the interview guide and study objectives, including food preparation, food allocation, food sharing, and caregiving practices. These domains served as initial analytical anchors rather than predetermined themes. Transcripts were then coded line by line to identify practices, experiences, and meanings described by participants. Codes were developed iteratively and refined as analysis progressed, allowing the thematic structure to evolve in response to the data.

Coding was led by the first author and principal investigator (MAS) using NVivo 15.2, with regular discussions among members of the research team. The coding structure and emerging themes were reviewed by a senior researcher (SC) to strengthen analytical rigour and ensure that interpretations remained grounded in participants’ accounts. Codes were compared across interviews and grouped into subthemes, which were subsequently organised into broader themes capturing both shared patterns and contextual variation. Finally, the themes were interpreted in relation to existing literature, with moral economy theory used as an interpretive lens to deepen understanding of the study findings.

### 2.4 Ethics statement

Ethical approval for this study was obtained from the Human Ethics Office of the Australian National University (H/2025/0121), the Northern Territory Department of Health and Menzies School of Health Research Human Research Ethics Committee (NT HREC; 2025-5085), and the Instituto Nacional de Saúde de Timor-Leste (INSP-TL; 386/INSP-TL/UEPD-AL/IV/2025). Written informed consent was obtained from all participants prior to participation. A copy of the participant information sheet and consent form is provided in Supplementary File 2.

## 3 Results

### 3.1 Participant demography

A total of 34 mothers with caregiving responsibilities for children in their households participated in the study, recruited from three municipalities: Dili (*n* = 14, 41.2%), Ainaro (*n* = 10, 29.4%), and Ermera (*n* = 10, 29.4%). Participants had a median age of 35 years (range: 18–45 years). Most participants had completed secondary education (*n* = 25, 73.5%), followed by primary education (*n* = 6, 17.6%) and university-level education (*n* = 3, 8.8%). Households were generally large, with a median household size of 9 members (range: 5–15) and a median of 4 children per household (range: 1–8). Regarding the age composition of children within households, 11 households (32.4%) included at least one child aged <2 years, 23 (67.6%) included at least one child aged 2–5 years, 27 (79.4%) included at least one child aged 6–12 years, and 23 (67.6%) included at least one child aged 13–18 years. These age-group categories were not mutually exclusive, as individual households could include children from multiple age groups.

Household income reflected diverse formal and informal livelihood arrangements. Approximate monthly household income was US$100–500 for 18 households (52.9%), above US$500 for nine (26.5%), and below US$100 for seven (20.6%). Income sources were diverse and often overlapping, including salaried or regular paid employment (*n* = 23, 67.6%), small-scale trading (*n* = 16, 47.1%), farming and the sale of agricultural produce or livestock (*n* = 10, 29.4%), government allowances or pensions (*n* = 7, 20.6%), and remittances or overseas earnings (*n* = 4, 11.8%).

Eleven households (32.4%) had one earning member, 14 (41.2%) had two, and nine (26.5%) had three or more earning members. Working household members were engaged in government, public-sector, or local-authority work (*n* = 18, 52.9%), private-sector, non-governmental organisation, or other regular paid employment (*n* = 16, 47.1%), small-scale trade, food sales, or other informal sales (*n* = 13, 38.2%), agriculture or livestock-related work (*n* = 7, 20.6%), manual labour, construction, or driving (*n* = 6, 17.6%), and overseas or seasonal work (*n* = 4, 11.8%). Income-source and occupational categories were not mutually exclusive, as households could report multiple income sources and occupational activities.

### 3.2 Overview of thematic findings

The thematic analysis generated five interrelated themes comprising ten subthemes describing the organisation and maintenance of household food practices in Timor-Leste. The five themes were: (1) women’s roles and authority in household food systems, (2) food allocation and protection within the household, (3) food sharing and social regulation beyond the household, (4) income instability creates chronic precarity and periodic food constraint, and (5) knowledge transmission and dietary practices.

**Figure 2:**
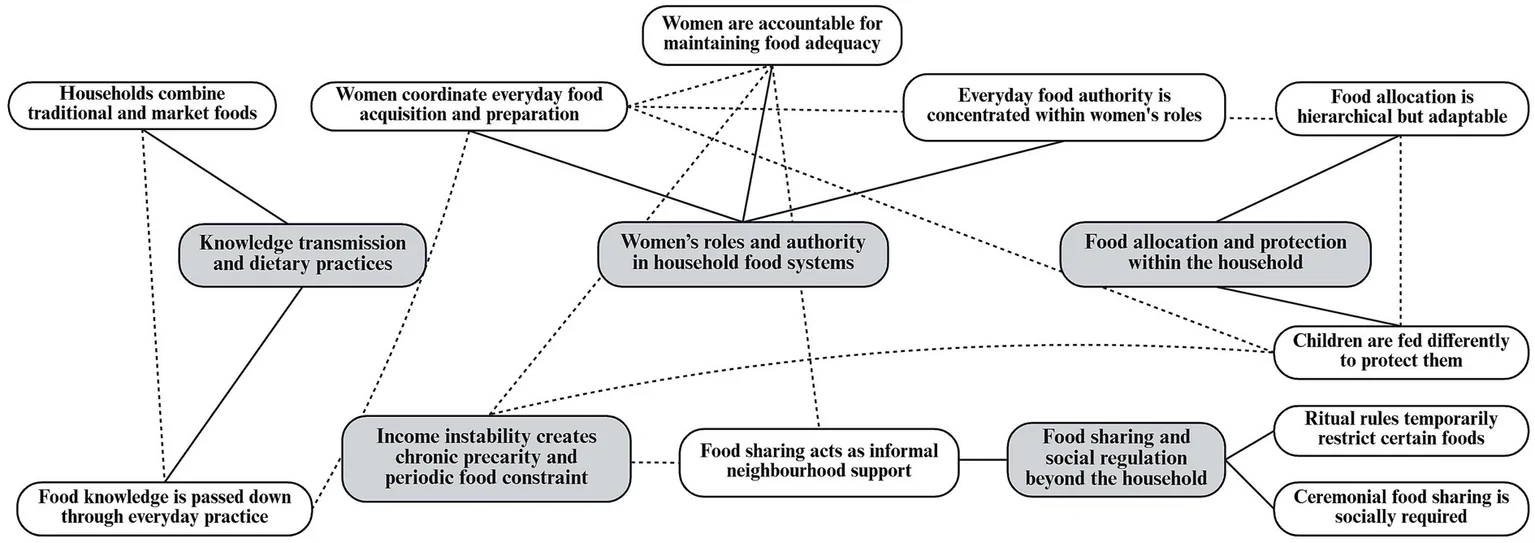
Thematic map of household food governance in Timor-Leste. Themes (light grey) and subthemes (white) are linked hierarchically (solid lines) and conceptually (dotted lines indicate conceptual relationships).

The thematic map (Figure 2) illustrates that these themes were analytically distinct but conceptually connected. Women’s authority in everyday food work intersected with patterns of household food allocation and child protection and with household responses to economic instability. Economic precarity was linked to both intra-household distribution and food sharing practices beyond the household. Intergenerational knowledge transmission informed dietary practices and intersected with everyday food coordination and adaptation. Together, the thematic map indicates that household food practices operated as an interconnected system, with governance, distribution, economic conditions, and cultural continuity operating in relation to one another rather than as discrete domains.

### 3.3 Women’s roles and authority in household food systems

#### 3.3.1 Women coordinate everyday food acquisition and preparation

Across households, women coordinated the everyday acquisition and preparation of food, including shopping, cooking, and serving meals. Men and other family members also contributed to food procurement, particularly when transport or physical strength was required, but women remained central to coordinating these activities. As one participant explained:

*“I am the one who usually buys the food, but sometimes my husband and children, when they go out, they also buy it, but I am the one who often do [sic] it” (Dili-Participant 3).*

Even where husbands were recognised as household heads, practical control over cooking and serving remained with women. Men’s participation was frequently linked to physical strength rather than oversight:

*“In our household, the head of the family (my husband) is the one who usually buys food for our household. I usually cook and serve food in our house.” (Ainaro-Participant 8) “For heavy foods that require strength, such as rice, my husband is usually the one who buys them because he rides a* motorbike, *and I am responsible for buying less heavy foods such as vegetables and others.” (Dili-Participant 8).*

In extended households, coordination was shared across generations of women rather than transferred to men:

*“We take turns helping each other. Sometimes, my grandmother is the one who goes shopping, sometimes my mother, and sometimes myself.” (Ainaro-Participant 2).*

A distinction emerged between participation in food procurement and the coordination of everyday food work. Although men and other household members sometimes purchased or transported food, women coordinated acquisition, preparation, and serving across daily household routines.

#### 3.3.2 Women are accountable for maintaining food adequacy

Ensuring food availability was framed as a responsibility rather than as optional domestic work. Women’s caregiving responsibility was closely associated with their ability to maintain meal continuity, even when resources were limited. One participant recalled childhood expectations:

*“When I was a child there was always food, when I saw there was no food, I scolded my mother and my sisters until my father came to scold my mother, and my mother made food for us to eat.” (Dili-Participant 9).*

Even when men contributed financially, accountability for meal provision remained attached to women. Substitution occurred only temporarily:

*“When I am sick, my husband can change [sic] me to go to buy food and cooks [sic] for our household.” (Ainaro-Participant 6).*

Under financial constraint, women described rationing and sequencing meals to maintain continuity:

*“We feel it’s a bit difficult because of the lack of money, we only buy the cheaper vegetables. When we buy them, I divide them immediately for the next day or the day after.” (Dili-Participant 10).*

These narratives indicate that maintaining food adequacy was embedded within caregiving identity, with women held accountable for meal continuity even under illness or financial constraint. Meal provisioning therefore represented a caregiving obligation rather than simply a reflection of resource availability.

#### 3.3.3 Everyday food authority is concentrated within women’s roles

Although decision-making was framed as mutual, everyday coordination of food acquisition, preparation, and allocation remained within women’s responsibilities. Financial provision and physical labour did not automatically confer governance over food practices.

In male-absent households, responsibility shifted across generations of women without altering expectations of provision:

*“Because now there is no adult male at home. So, my grandmother, my mother and I have taken on the responsibility of buying foods [sic] for our household.” (Ainaro-Participant 2).*

Where men were present, decision-making was described as jointly discussed:

*“The strategies we use are always decided together by my husband and me” (Ermera-Participant 4).*

Overall, shared household discussion and everyday food governance operated at different levels. Participants described decisions as jointly discussed, while practical coordination of food acquisition, preparation, and allocation remained concentrated within women’s roles. This food-related authority represented domain-specific operational authority arising from women’s responsibility for maintaining everyday food provision and should not necessarily be interpreted as broader household power or empowerment. Household food governance therefore emerged through caregiving responsibility alongside formal household hierarchy and income control.

### 3.4 Food allocation and protection within the household

#### 3.4.1 Food allocation is hierarchical but adaptable

Food allocation followed a recognised hierarchy yet remained flexible under pressure. Serving order was commonly structured by age, gender, and income contribution, with husbands or household heads frequently eating first. In some households, older family members were prioritised, and age-based sequencing was evident among children, with older members or older children eating before younger ones. This sequence signified respect and status within the household.

*“My husband usually eat [sic] first. Because he is the one who find [sic] the food and sometimes pay [sic] for it.” (Ermera-Participant 7).*

*“Once the meal is cooked, I first serve my grandmother and my mother, and then all of us eat together.” (Ainaro-Participant 2).*

However, allocation was not rigid. During economic constraint, prioritisation shifted:

*“During difficult times, we always give priority to children who need to eat.” (Dili-Participant 14).*

Allocation reflected a negotiated balance between social hierarchy, intergenerational respect, and caregiving obligation, where recognised status shaped serving order but vulnerability reshaped distribution during periods of scarcity.

#### 3.4.2 Children are fed differently to protect them

Within the wider system of food allocation, feeding young children functioned as a protected and carefully managed practice. Children were not simply given the same food as adults. Instead, meals were adjusted based on age, growth stage, and what the child could tolerate.

*“Starting from six months old, we begin giving porridge until they reached [sic] one year of age.” (Ainaro-Participant 2).*

Mothers described actively changing meals when children refused certain foods, showing that child feeding involved ongoing judgement rather than routine repetition of household dishes.

*“They would not eat porridge. So, I stopped giving them porridge and replaced it with formula. The decision to serve soft rice was my own idea.” (Dili-Participant 8).*

Control over children’s diets was consistently held by mothers. Decisions about what children should eat were described as their responsibility, even within households where food decisions were otherwise shared.

*“The decision regarding what food to give my children is made solely by me.” (Ainaro-Participant 2).*

During times of financial difficulty, this protection became more visible. Adults described reducing their own intake so that children would not go hungry.

*“Adults can eat or not eat, but children must not go hungry.” (Ermera-Participant 10).*

Child feeding operated as a protective buffering mechanism within the household food system. Participants described adults reducing their own consumption to protect children, but the interview data did not consistently distinguish whether this reduction differed between mothers and fathers. While everyday serving orders reflected status and contribution, children’s meals were organised around need and vulnerability. Household distribution, therefore, combined role-based hierarchy with deliberate protection of those considered most at risk. These findings indicate that child feeding is actively protected within households but remains vulnerable to economic instability and resource constraints.

### 3.5 Food sharing and social regulation beyond the household

#### 3.5.1 Food sharing acts as informal neighbourhood support

Food sharing beyond the household operated as a routine social system rather than occasional generosity. Food was shared regularly between neighbours and relatives, shaped by kinship, proximity, and awareness of each other’s needs.

*“If the neighbour has food, we eat with them. If we have vegetables or food, we also share it with our neighbours.” (Dili-Participant 4).*

Participants described sharing as reciprocal rather than charitable, with exchange expected to flow both ways.

*“Sharing works like this: I give to them, they give to me.” (Dili-Participant 10).*

These exchanges functioned as an informal system of redistribution, enabling households to borrow food with the expectation that it would later be returned. Even small quantities were considered appropriate to share, reflecting social norms that prioritised collective support during periods of need. In some situations, sharing was socially required, particularly during culturally significant events.

*“When our buffalo dies, we have to share it with the neighbours; we are not allowed to eat it alone.” (Ainaro-Participant 5).*

These practices operated through close networks of kinship and neighbourhood relations, where reciprocity created mutual expectations to share food when resources became available. Food sharing therefore functioned as an informal system of redistribution that helped households manage short-term shortages while also reflecting social obligations that could make sharing expected rather than entirely voluntary.

#### 3.5.2 Ceremonial food sharing is socially required

Ceremonial and religious events represented a distinct form of food redistribution that was public, structured, and socially regulated. Unlike everyday food sharing between neighbours, participation in these events required households to contribute specific food items or animals according to the type of ceremony and their perceived social responsibility within the community.

*“If there is a cultural ceremony, we have to bring a sheep, cow, or pig.” (Ermera-Participant 2).*

Participants described these contributions not as voluntary generosity but as socially recognised obligations. Food brought by different households was pooled, prepared collectively, and consumed together, reflecting the communal organisation of ceremonial meals.

*“In the community, when there is a religious event, each resident cooks food to be put together and eaten together.” (Dili-Participant 7).*

In this context, food circulated through collective preparation and shared consumption, reinforcing participation and visibility within the community. Redistribution also extended beyond the communal meal, as leftover food was divided and sent back to households, allowing participation to be recognised even after the event itself.

Despite their social importance, participants acknowledged that repeated ceremonial obligations could create financial pressure for households with limited resources.

*“After attending several of these events, our money may be exhausted.” (Ermera-Participant 8).*

Ceremonial food sharing, therefore, functioned simultaneously as a mechanism of social cohesion and a system of collective obligation. Participation reinforced kinship ties, community identity, and mutual recognition, while also reflecting the economic tensions that households navigated in maintaining socially expected forms of contribution.

#### 3.5.3 Ritual rules temporarily restrict certain foods

Participants generally described everyday food practices as flexible, with few permanent food prohibitions. However, this flexibility coexisted with temporary ritual restrictions that regulated when certain foods could be consumed. These restrictions were linked to harvest cycles, ancestral customs, and ceremonial timing. The most frequently mentioned example was the *sau batar* ritual, a traditional thanksgiving and corn harvest ceremony practised by agricultural communities in Timor-Leste.

*“All vegetables cannot be eaten before ‘sau batar.’ Once ‘sau batar’ is reached, everything can be eaten freely.” (Dili-Participant 8).*

Participants explained that these practices were passed down by older generations and followed as inherited obligations.

*“This custom was taught to us by older generations… everyone followed it.” (Ermera-Participant 2).*

In some households, additional lineage-based restrictions were observed, although adherence was sometimes selective. Rather than permanently prohibiting specific foods, these practices regulated consumption through ritual timing and inherited authority. Outside ceremonial periods, food practices remained practical and adaptable, while ritual rules temporarily structured when certain foods could be eaten. These accounts show how everyday eating was organised through culturally embedded norms and ceremonial timing.

### 3.6 Income instability creates chronic precarity and periodic food constraint

Food access was shaped by income stability rather than physical availability. Participants repeatedly explained that markets had food, but purchasing depended on having money at the right time.

*“We never encounter problems for lack of food because everything depends on money.” (Ainaro-Participant 10).*

Despite many households reporting some form of salaried or regular paid employment, participants frequently described household income as uncertain because earnings often depended on multiple informal, seasonal, or fluctuating sources. Income was commonly obtained through selling vegetables, small trade, farming, or casual work, and participants described reliance on additional informal or variable income sources.

*“There is nobody in our household who has a salaried job. We earn money from selling items…. There are two objectives for growing vegetables, including for our daily consumption and for sale when it is available in big quantities.” (Ainaro-Participant 4).*

Households relied on multiple small and fluctuating income sources, where food production served both subsistence and income purposes and cash earnings varied from day to day.

*“Our income is not fixed, because some days we do not earn anything.” (Ermera-Participant 3).*

Persistent reliance on unstable income created chronic economic precarity, while periodic interruptions in earnings produced more immediate food constraints. During these periods, eating patterns changed quickly:

*“Instead of eating three times a day, we decreased it to twice or even once.” (Ainaro-Participant 2).*

Scarcity was therefore experienced as contraction rather than absolute absence, with periodic interruptions in earnings triggering immediate reductions in meal frequency, portion size, or dietary diversity. Food adequacy consequently expanded or contracted in line with income flow.

### 3.7 Knowledge transmission and dietary practices

#### 3.7.1 Food knowledge is passed down through everyday practice

Food knowledge was learned through everyday household practice rather than formal instruction. Participants described learning through observation, participation, and repetition while assisting mothers and older women in food preparation.

*“My knowledge of cooking comes mainly from my mother. She taught me how to cook, how to prepare rice, and how to make certain dishes and sweets. In those situations, people do not formally teach you, but you observe how food is prepared and learn by watching and following what others do.” (Ermera-Participant 5).*

Girls were expected to assist with cooking from a young age, with skills developing gradually through participation in routine household work rather than through formal instruction. Cooking skills were viewed as essential for managing everyday household life, and passing on this knowledge ensured continuity of family food practices across generations. Although participants acknowledged generational change, particularly the time required for traditional preparation, culinary authority remained anchored in knowledge passed down from older generations.

*“I never learned to cook from television or YouTube” (Ermera-Participant 6).*

Digital media was occasionally mentioned but was not described as a primary source of learning. Instead, participants emphasised knowledge transmitted through parents and grandparents, showing how everyday participation in food preparation sustained culinary knowledge across generations despite gradual social change.

#### 3.7.2 Households combine traditional and market foods

Daily diets reflected a combination of home-grown staples and purchased foods. Most households described meals centred on cassava, corn, taro, pumpkin, beans, and other garden crops, while rice and some processed foods were obtained from local shops.

*“Rice, taro, corn, pumpkin (fehukropa), and cassava, these are the only foods that come from the garden.” (Ainaro-Participant 1).*

Locally grown staples remained central to daily eating because they were affordable, accessible, and embedded in routine household food practices. Purchased foods, particularly processed items, were used more selectively and were sometimes viewed with caution.

*“They usually want sausages, but we do not buy them, because sausages have a lot of chemicals.” (Ainaro-Participant 8).*

Participants explained that children’s preferences and convenience occasionally influenced food purchases, but cost and health concerns limited the regular use of processed foods. At the same time, some households described gradual changes in food production, particularly the decline of homegrown rice.

*“Now that we no longer grow rice ourselves, we have no choice but to buy it from shops.” (Ainaro-Participant 4).*

Market dependence was therefore described as a practical adjustment rather than a rejection of traditional foods. As garden production declined, purchased rice became more common, reflecting broader changes in labour, land use, and household livelihoods. Daily diets thus combined traditional staples with selected market foods, illustrating how households adapted food practices to changing economic and production conditions.

## 4 Discussion

This study examined everyday household food practices in Timor-Leste and their implications for household food security and child feeding. Rather than viewing food practices as individual dietary behaviours, the findings show that food acquisition, preparation, allocation, and sharing are embedded within social relations, caregiving responsibilities, and household economic constraints. Women played central roles in coordinating everyday food work and maintaining food adequacy under conditions of income instability. These practices were relevant to child feeding, with households prioritising children during periods of scarcity while adjusting meal frequency, food choices, and distribution in response to limited resources. Food insecurity was experienced and managed not only in relation to economic limitations but also through household decision-making, caregiving roles, and systems of reciprocity within communities. Overall, these findings highlight household processes relevant to food security and potentially to child nutrition vulnerability in low-resource settings.

### 4.1 Women’s caregiving authority in everyday food work

Across households, women were positioned as the primary custodians of everyday food work, responsible not only for purchasing, preparing, and allocating food, but also for judging what was adequate, appropriate, and fair under economic constraint. While gendered divisions of food labour are well documented in the literature (9), this study demonstrated that women’s food work was framed less as routine domestic labour and more as a responsibility. Women’s authority over everyday food decisions did not necessarily correspond with control over household income. This finding aligns with moral economy theory, which emphasises that food provisioning is shaped by shared expectations of responsibility, care, and obligation (10, 12).

Food work was also organised collectively across generations, with daughters, daughters-in-law, and grandmothers contributing according to availability and capacity. This finding highlights provisioning as a collective and adaptive practice rather than a fixed division of labour, consistent with kinship-based organisation of everyday cooperation and knowledge circulation (20). This reinforces caregiving continuity without fundamentally altering broader gendered expectations of responsibility.

### 4.2 Household authority, hierarchy, and negotiated power

Food-related decision-making emerged as a negotiated and situational process shaped by household hierarchy, caregiving roles, and economic pressure. While women frequently held operational authority over decisions concerning food access, allocation, and coping strategies, this authority was neither absolute nor unilateral. Instead, decisions were commonly reached through discussion and agreement, reflecting concern for household cohesion and care under constraint.

A key finding is that control over income did not automatically mean control over food decisions. Financial provision and everyday adequacy operated as distinct spheres of responsibility. This aligns with research conducted in the Philippines showing that household authority is contingent and negotiated through caregiving identity rather than exercised solely through economic dominance (21). This distinction highlights how economic and caregiving domains operate in parallel but are not interchangeable in shaping food-related authority.

The coexistence of hierarchy and cooperation reflects evidence that fairness is often understood as prioritising those with greater need rather than treating all household members equally (22). In Timorese households, food allocation involved prioritisation of household heads, older family members such as grandmothers, and children in different contexts, while eating together in some households indicated social cohesion. These patterns suggest that meal sharing functions as a marker of belonging and mutual recognition even where inequalities persist (11).

The absence of adult men due to migration, study, or employment in some households reshaped labour distribution but did not fundamentally alter gendered expectations. In male-absent households, women were primarily responsible for food preparation and also for food purchasing and acquisition, highlighting that food security is shaped by labour availability as well as income (23, 24). However, taking on these responsibilities did not automatically increase symbolic power. Expanded responsibility did not necessarily translate into proportional increases in decision-making authority.

### 4.3 Food allocation and feeding children

Food allocation followed socially recognised patterns shaped by age, gender, and perceived contribution. However, these arrangements were flexible rather than rigid. During periods of scarcity, children were consistently prioritised.

Moreover, this flexibility reflected moral negotiation rather than strict hierarchy, where fairness was defined as equity (allocating according to need) rather than equal portions, consistent with moral economy theory (10, 12, 25). Similar findings from other contexts show that food distribution is an ethical and relational practice shaped by shared expectations of responsibility and dependence (22). Accordingly, prioritising children represented a morally legitimate reordering within caregiving relationships rather than a departure from social norms.

Feeding young children was described as a core commitment closely tied to caregiving identity. Decisions about what children ate and how food was allocated were shaped by age, food availability, and household economic constraints, with mothers often retaining primary responsibility. Caregivers described reducing their own intake, substituting foods, or adjusting meal patterns to shield children from hunger. These adjustments illustrate how vulnerability to inadequate child feeding was actively managed within households during periods of economic constraint. Moreover, emotional distress when children lacked food shows that food insecurity was experienced both materially and emotionally. Overall, feeding children held central importance within caregiving identity.

### 4.4 Intergenerational knowledge transmission and dietary adaptation

Food knowledge was transmitted primarily through intergenerational relationships, particularly from mothers and grandmothers to younger women, through observation and everyday practice rather than formal instruction. Teaching daughters was framed as preparation for future caregiving roles, indicating that learning food practices involved developing responsibility within the household (26). Through daily practice, skills and judgement were carried across generations, helping to sustain household food practices under changing household conditions.

Although gardens supplemented diets, households remained reliant on markets, reflecting mixed livelihood systems in which subsistence, cash income, and social exchange coexist (20, 27, 28). Dietary transition was framed as pragmatic adaptation rather than cultural rejection, as traditional staples were combined with purchased foods through everyday negotiation shaped by labour constraints, generational preferences, and evaluation (27, 29). Traditional staples functioned as anchors of stability, while market foods were incorporated selectively in response to labour constraints, generational preferences, and cash availability. These patterns illustrate how stability and change coexist within household food systems, with traditional knowledge anchoring practices while market integration introduces adaptive flexibility. These findings also highlight an apparent tension between the cultural continuity of food practices and persistent nutritional vulnerability. Intergenerational knowledge and attachment to traditional foods may sustain household food systems as families adapt to changing livelihoods and market integration, but these practices cannot necessarily overcome the economic and resource constraints that limit dietary quantity and diversity. Tradition may therefore provide continuity in how food is prepared, shared, and valued without necessarily ensuring nutritional adequacy when households face persistent material constraints.

### 4.5 Social regulation, reciprocity, and constraint

Across households, food practices were embedded within social relationships in which sharing, restriction, access, and dietary change were governed by socially recognised obligations rather than individual choice alone. Food rules were not fixed taboos, but situational norms activated through lineage, seasonality, or ritual context, enacted when boundaries were salient rather than imposed on daily diets (25). Together, these findings suggest that household food systems in Timor-Leste function as relational governance structures through which care, obligation, and adaptive practices regulate food adequacy under conditions of economic volatility. Viewed through a moral economy lens, reciprocity represents not simply resource exchange but a socially embedded expectation through which households negotiate mutual responsibility and food access during periods of constraint. Reciprocity further operated as an informal redistribution system that stabilised food access beyond individual households.

Income instability also shaped food adequacy through the interaction of chronic economic precarity and periodic food constraint. Persistent reliance on informal, seasonal, or fluctuating earnings created underlying vulnerability, while short-term interruptions in cash flow led to immediate changes in meal frequency, portion size, and dietary diversity. Similar coping responses, including eating cheaper foods and reducing meal size and frequency, have previously been documented in Timor-Leste (30). Seasonal fluctuations in food security and dietary diversity, together with consumption-related coping through changes in dietary quantity, quality, and frequency, have also been described in other resource-constrained settings (31). The findings of this study extend this evidence by showing how food adequacy may repeatedly expand and contract with income flow, creating recurrent episodes of constraint even where food remains physically available in markets.

Although this study was not designed to formally compare urban and rural settings, several common patterns were evident across the three municipalities. Women’s central role in everyday food management, the prioritisation of children during periods of scarcity, reciprocal food sharing, and the effects of income instability were described across both urban and rural settings. Some contextual differences were also apparent. Household food production and reliance on garden crops were more evident in rural settings, whereas reliance on purchased food and cash availability was more prominent in the urban setting. Despite these differences, households across settings adapted food acquisition, allocation, and meal practices in response to available resources, suggesting that caregiving responsibility and economic constraint were common influences on everyday food management.

### 4.6 Implications for food security interventions

This study has implications for public health and nutrition programmes in low-resource settings by advancing a conceptual understanding of household food systems in Timor-Leste as gendered and relational governance structures through which food-related practices are coordinated, negotiated, and regulated under economic and ecological volatility. Authority over food operated through caregiving legitimacy rather than income dominance; allocation combined hierarchy with protective flexibility; and reciprocity functioned as informal redistribution across households. These findings complement approaches to food security that emphasise household income, supply, or behaviour by highlighting how everyday food adequacy is also organised through negotiated authority, obligation, and adaptive buffering within volatile livelihood contexts. While grounded in Timor-Leste, these findings have broader relevance for low- and middle-income settings where food practices are shaped by informal economies, gendered labour, and reciprocal social systems.

In practice, nutrition and food-security interventions that focus narrowly on individual behaviour change risk overlooking how food decisions are socially structured. Effective interventions should recognise women’s central roles in everyday food management, the priority given to children, and the importance of reciprocal networks in stabilising access. Programmes that support income stability and work with existing caregiving roles may be better aligned with household food practices in low-resource settings.

### 4.7 Limitations

This study was conducted in three municipalities (Dili, Ainaro, and Ermera) and does not capture the full geographic, ecological, and cultural diversity of food practices across Timor-Leste. Although participants were recruited from diverse settings of urban and rural communities, this study was not designed to systematically compare differences by municipality or setting. Therefore, the findings should be interpreted as context-specific rather than representative of the entire country. The study included only mothers with caregiving responsibilities for children in their households because of their direct involvement in everyday food preparation, feeding, and household food management. Consequently, the findings reflect women’s perspectives and may not capture how men or other household members understand food-related authority and decision-making. Additionally, the exclusion of women aged over 45 years may limit perspectives on intergenerational food knowledge and practices. Therefore, future research including men, other household members, and older women could provide a more comprehensive understanding of household food practices and power relations.

## 5 Conclusion

This study examined everyday household food practices in Timor-Leste and their implications for household food security and child feeding. The findings suggest that household food practices operate as relational systems of governance in which food acquisition, allocation, and sharing are organised through gendered caregiving authority, negotiated hierarchy, reciprocal redistribution, and adaptive responses to income instability. Women emerged as central coordinators of household food systems, while allocation practices combined recognised hierarchy with protective prioritisation of children during periods of constraint. Income volatility reduced food quantity and variety, while caregiving authority organised how limited resources were prioritised and distributed within households. Recognising food security as a socially organised process of decision-making rather than solely a function of resource availability has important implications for intervention design. Programmes that recognise women’s operational authority, support income continuity, and work within existing systems of reciprocity may be better aligned with the social organisation of household food practices.

## Data Availability

The data that support the findings of this study consist of qualitative interview transcripts and are not publicly available due to ethical and confidentiality considerations. De-identified excerpts are included within the article. Additional data may be made available from the corresponding author upon reasonable request, subject to approval by the relevant ethics committees and data-sharing agreements.
