# Supplementary material for: Household food practices and implications for food security and child feeding in Timor-Leste
Source: Front Public Health. 2026 Sep 7;14:1852471. doi: 10.3389/fpubh.2026.1852471 (PMC13614097; doi:10.3389/fpubh.2026.1852471)
Supplement: Supplementary file 1 [file supplementary_file_1.docx]

**Supplementary File 1.** Semi-structured interview guide used in qualitative interviews in Timor-Leste

This interview guide was developed for the broader qualitative study exploring household food practices in Timor-Leste. Different sections of the guide informed separate thematic analyses presented in related publications.

**Section A: Background information**

1. Municipality:
2. Administrative Post:
3. Village:
4. Gender of the interviewee: [Male / Female / Other]
5. Age of the interviewee: [18-24 / 25-34 / 35-44 / 45-54 / 55-64 / 65+]
6. Highest education level: [Illiterate / No formal education / Primary / Secondary / University]
7. Number of household members: [Open-ended]
8. Number of children in the household: [Include age ranges: <2 years/ 2-5 years, 6- 12, 13-18 years]
9. Source of household income: [Open-ended or common options]
10. Earning members in the household: [Open-ended]
11. Household's approximate monthly income: [Range options, e.g., <$100, $100–$500, >$500]
12. Number of working members and their occupations: [Open-ended]

**Section B: Household Food Practices – Choices and Preparation**

**Food Sourcing and Shopping**

1. Do you grow your own food? If yes, which foods?
2. Who usually buys food for the household?
3. Where do you get food during:

- The wet season:
- The dry season:

4. If access becomes difficult, how do you manage (e.g., asking neighbours, bartering, community support)?

Shopping/sourcing food table:

| *Shopping/sourcing food* | *How often* | | *Types of food* | *Who buys* | *Distance from home* | *Transport used* | *Shopping preference* | | | |
| --- | --- | --- | --- | --- | --- | --- | --- | --- | --- | --- |
|  | *Wet season* | *Dry*  *season* |  |  |  |  | *Fresh* | | *Frozen* | |
|  |  |  |  |  |  |  | *Wet* | *Dry* | *Wet* | *Dry* |
| *Supermarket* |  |  |  |  |  |  |  |  |  |  |
| *Local market* |  |  |  |  |  |  |  |  |  |  |
| *Home garden* |  |  |  |  |  |  |  |  |  |  |
| *Fishing* |  |  |  |  |  |  |  |  |  |  |
| *Bartering* |  |  |  |  |  |  |  |  |  |  |
| *Other* |  |  |  |  |  |  |  |  |  |  |

**Sharing Food with Others:**

5. What are the main sources of food specifically for children under five in your household? (e.g., homegrown, purchased, donated, shared by relatives)
6. Are foods for children under five prepared differently from others in the household? If yes, how? (e.g., softer texture, boiled separately, no spices, hygienic precautions)
7. How food is shared in your family —
   a. In normal times? For instance, who usually eats first, or is food equally shared among all members?

b. When food is limited? For instance, are certain members (e.g., elders, children, or men) prioritized for meals when food is limited?

8. Do you share food with others in the community? If yes:
a. With whom?
b. What foods are shared?
c. On what occasions or reasons (e.g., weddings, cultural events, religious celebrations)?

**Passing Down Food Knowledge:**

9. Who teaches food-related skills in your family? [Specify roles such as parents, elders, or others.]
10. What knowledge is passed on? [e.g., recipes, preparation methods, food safety practices, cultural traditions, or modern cooking techniques from external sources such as workshops or online videos in YouTube.]
11. How is it shared? [e.g., verbal teaching, observation, participation in activities, or social media platforms.]
    a. Do children learn traditional food skills? If yes, how? If not, why not?

**Section C: Food Preparation and Consumption:**

1. Which type of kitchen do you have? [Private/Shared]
2. Is water for cooking or washing always available? If not, what challenges do you face?
3. Where does your household water come from? (e.g., well, river, shared tap)
   a. If water is stored, is it covered? [Yes/No]
4. Is meat and fish cooked as soon as it is purchased or killed? [Yes/No/Sometimes]
   b. If not, how is it stored?
5. Who is usually responsible for preparing and serving food in your family?
6. What foods are most consumed in your household?

(Please complete the table below):

| Food name | Category  (Local/Imported Modern) | Frequency of cooking per week | Who eats it  (Child/ Adult/ Elderly) | Ingredients | Refrigerated (Y/N) |
| --- | --- | --- | --- | --- | --- |
| *Eg.Fish with veg* | *Local* | *3* | *Everyone* | *Fish & Veg* | *Fish – Y Veg - N* |
| *Eg.Sausage and Rice* | *Imported* | *21* | *Everyone* | *Sausage and Rice* |  |
| Eg.Tempeh/ tofu |  |  |  |  |  |
|  |  |  |  |  |  |
|  |  |  |  |  |  |
|  |  |  |  |  |  |
|  |  |  |  |  |  |

7. Are there foods only eaten on special occasions or avoided (taboo)? If yes, please describe and explain why.

**Section D: Food Preservation and Storage**

1. How do you preserve food at home? (e.g., drying, refrigerating, salting)
2. What challenges do you face when storing food?
3. Do cultural beliefs affect how food is preserved or eaten?
4. Do you store food differently in the wet vs. dry season? If yes, how?
5. Are there foods kept longer for cultural or ritual reasons?
6. Are leftovers used over several days? If yes, how are they stored?
7. Are some foods prepared or stored differently based on who eats them (e.g., men vs. women)?

**Section E: Traditional Practices and Community Support**

1. What traditional Timorese food practices (e.g., preparation, storage) are important to you? Why?
2. How are traditional foods usually prepared in your community?
3. What challenges do you face in continuing these practices?
4. In hard times, do you receive help from others (e.g., neighbours, leaders, groups)? If yes, what kind of support?

**Section F: Open-Ended Feedback**

Is there anything else you’d like to share about food habits, challenges, or beliefs in your household or community?
