# Supplementary material for: Household food practices and implications for food security and child feeding in Timor-Leste
Source: Front Public Health. 2026 Sep 7;14:1852471. doi: 10.3389/fpubh.2026.1852471 (PMC13614097; doi:10.3389/fpubh.2026.1852471)
Supplement: Supplementary file 2 [file supplementary_file_2.docx]

**PARTICIPANT INFORMATION SHEET**

**THIS IS FOR YOU TO KEEP**

**Researcher:**

**Researcher: Dr. Lesy Careca Atok, Research** Coordinator, Menzies School of Health Research, Timor-Leste

**Telephone:** +670 75760534

**Email:** [lesy.atok@menzies.edu.au](mailto:lesy.atok@menzies.edu.au)

and

**Researcher:** Dr Md Abu Sayeed, The Australian National University (ANU) in Canberra, Australia.

**Telephone:** +61480494402
**Email:** abu.sayeed@anu.edu.au

**Title of Study:** Exploring the influence of cultural norms, practices, and community factors on food choices, preparation and food storage in the Timorese community

Dear Participant,

We are inviting you to take part in a study. We want to learn how people’s beliefs, practices, and community life affect the food they eat, how they cook it, and how they store it in Timor-Leste. This information will help us develop better ways to make food safer and improve health.

**What is the research about?**

We want to understand how your culture and community affect your food habits, such as the foods you choose, how you prepare your food, and how you store your food, in different parts of Timor-Leste. This will help us create health interventions that are suitable for communities in Timor-Leste.

**Who is conducting this research?**

This research is being done by a team of experienced researchers from Menzies School of Health Research, led by Salvador Amaral.

**Why am I being invited to participate?**

You have been invited because you help with preparing food in your family. Your knowledge can help us learn more about local food habits.

**What will I be asked to do?**

If you agree to join, you will take part in a face-to-face interview with a researcher. We will ask questions about the food you and your family eat, how you prepare and store it, as well as any tradition that guide how you do this. The interview will take about 30 to 45 minutes. We will record the interview with your permission. We will meet at a time and place that works best for you.

**Are there any risks associated with participating in this research?**

Being in this study is your choice. Everything you say will be kept private, and your name will not be shared in any report. You can skip any questions you do not want to answer.

**What are the benefits?**

Your participation will help us to learn how to make food safer and healthier in Timor-Leste. The information from this study will help to develop effective public health interventions. To thank you for your time, will be give you some phone credit or a nutritional package.

**Can I withdraw from the research?**

Yes, it is completely your choice to take part. You can say no or stop at any time. If you decide to stop, your information will not be used.

**What will happen to my information?**

Everything you share with us will be kept private. We will record the interview and then write it down. Your name will be removed, and only the research team will see your answers. Your information will be stored safely on a tablet and computer that are protected with a password in the Menzies School of Health Research in Timor-Leste. It will be kept in anonymised form for five years and then deleted. If you decide to stop, your information will not be used. Your name will never appear in any report or document.

**Complaints or questions**

If you have any concerns or complaints about the study, you can ask the study team at any time.

**Queries and Concerns:**

- Before you say yes to join this study, please make sure all your questions are answered. You can also talk to a doctor, nurse, or family member.
- Only sign the consent form if you feel comfortable and understand everything.
- If you later decide to leave the study, please let a member of the research team know.
- If during the study you need more information or if you have any problems with the study, please speak to one of the research team. You can also inform any representatives of the District Health Office (Telephone: +6703331176), or any member of the Timor-Leste Ministry of Health (Telephone: +6703331107).

**Contact Information**

For further information or to raise any concerns about the research, please contact:

Salvador Amaral

Email: [Salvador.amaral@menzies.edu.au]

Dr. Lesy Atok

Email: [lesy.atok@menzies.edu.au]

**Ethics Committee Clearance:**

This study has been approved by the Timor-Leste Instituto Nacional de Saúde Human Research Ethics & Technical Committee, ANU Human Research Ethics Committee, and Human Research Ethics Committee of Menzies School of Health Research. If you have any concerns or complaints about how this research has been conducted, please contact:

| Ethics Manager The ANU Human Research Ethics Committee The Australian National University Telephone: +61261253427 Email: [Human.Ethics.Officer@anu.edu.au](mailto:Human.Ethics.Officer@anu.edu.au) | Research Department  Instituto Nacional de Saúde Publica -Timor-Leste  Telephone: + +670 7806 2685  Email: uepd insp_tl uepd.insp.tl@gmail.com | Ethics Manager  Menzies ethics committee  Telephone: +61 8 8946 8600, email [NTHREC@menzies.edu.au](mailto:NTHREC@menzies.edu.au) |
| --- | --- | --- |

Thank you for considering participation in this important study. Your insights are invaluable to understanding and improving food safety and nutrition in Timor-Leste.

**FICHA INFORMASAUN PARTICIPANTE**

**IDA NE’E BA ITA ATU RAI**

**Peskizador:**

**Peskizador: Dr. Lesy Careca Atok,** Koordenador Peskiza, Menzies School of Health Research, Timor-Leste

**Telephone:** +670 75760534

**Email:** [lesy.atok@menzies.edu.au](mailto:lesy.atok@menzies.edu.au)

and

**Peskizador:** Dr Md Abu Sayeed, The Australian National University (ANU) in Canberra, Australia.

**Telephone:** +61480494402
**Email:** abu.sayeed@anu.edu.au

**Title of Study:** Esplora influensia husi normas kulturais, pratikas no fatores komunitarios ba opsaun ai-han, preparasaun no armazenamentu iha komunidade timorense

Karu partisipante,

Ita bo’ot hetan konvite hodi hola parte iha peskiza ida ne’e. Ami hakarak buka hatene oinsa fiar kultural sira, pratikas no komunidade nia moris influensia ema atu hili ai-han, oinsa seira prepara ai-han no oinsa sira armazena ai-han iha Timor-Leste. Informsaun ida ne’e sei ajuda ami hodi dezenvolve estratejia diak hodi dezenvolve ai-han seguru no nutrisaun.

**Peskiza ida ne’e konaba saida?**

Ami hakarak komprende oinsa kultura no kumunidade deside saida mak ema han, hanesan opsaun ai-han, oinsa sira tein, no oinsa sira armazena iha komunidade iha Timor-Leste nia laran. Ida nee sei ajuda atu kria programa de saude sira ne’ebe diak ba komunidade sira iha Timor-Leste.

**Se mak halao peskiza ida nee?**

This research is being done by a team of experienced researchers from Menzies School of Health Research, led by Salvador Amaral.

**Tanba sa mak konvida hau atu partisipa?**

Konvida mos ita boot tanba ita boot ajuda prepara ai-han iha ita boot nia uma laran. Ita boot nia esperiensia bele ajuda ami hodi komprende konaba pratikas ai-han lokal.

**Husu hau atu halo saida?**

Karik ita bo’ot aseita atu tuir, ita boot sei invitadu atu hola parte iha entrevista oin-ba-oin. Ami sei husu perguntas konaba ai-han neebe ita bo’ot no ita boot konsumu, oinsa ita bo’ot prepara no armazena ai-han sira, nune’e mos kultura ka tradisaun balun ne’ebe influensia ba hahalok sira ne’e. Entrevista sei dura minutu 30-45 no sei rekorda ba audio, ita ne’e sei halo iha tempu no fatin ne’ebe hakmatek ba ita bo’ot.

**Iha risku ruma ligadu ho bainhira partisipa iha peskiza ida ne’e?**

Partisipasaun iha estudu ida ne’e voluntariu, informasaun hotu ne’ebe ita boot informa mai ami sei asegura ninia privasidade no ita bo’ot nia naran sei la uza iha relatoriu ruma.Ita bo’ot bele la hatan pergunta balun ne’ebe ita bo’ot lakohi resposta.

**Benefisiu sira mak saida deit?**

Ita bo’ot nia partisipasaun sei ajuda ami komprende diak liu tan ai-han seguru no nutrisaun iha Timor-Leste.Informasaun sira husi peskiza ida ne’e sei ajuda hodi dezenvolve intervensaun saúde públika ne’ebe efetivu.

**Hau bele retira husi peskiza ?**

Sim, hola parte ida ne’e kompletamente voluntariu. Ita bo’ot bele para iha kualker tempu karik ita bo’ot deside atu sai, ita boot nia informsaun sei la uza.

**Saida mak sei akontese ba hau nia informasaun?**

All answers collected during the interview will be stored securely in a password-protected system. Only the research team will see the information. Audio recordings will be written down and stored safely Your name will not used in any reports or documents.

**keisas ka pesguntas**

Karik ita bo’ot iha preokupasaun ka keisas ruma konaba estudu, ita bo’ot bele husu ekipa peskiza iha kualker tempu.

**Perquntas no preokupasaun**

- Antes ita bo’ot decide atu tama ba estudu ne’e, asegura katak ita bo’ot nia pesguntas sira hatan hotu ona. Ita bo’ot mos bele koalia ba doutor ka enfermeru, ka membru familia ruma.
- Asina format konsetimentu sei halo depois de ita bo’ot satisfeitu ho ita bo’ot nia perguntas sira ne’ebe hatan ona. Sei mais tarde, deside atu abandona estudu, halo favor informa ba membru ekipa peskiza nian ruma.
- Karik durante estudu presiza informasaun barak liu tan ka ita bo’ot iha problema ruma ho estudu, bele koalia ba membru peskiza ruma. Ita bo’ot mos bele informa ba reprezentante ruma iha ofisina Servisu Municipal da Saude (Telephone: +6703331176), ka membru ruma iha Ministeriu de Saude Timor-Leste ( +6703331107)

**Informasaun ba kontaktu**

Atu hetan informasaun ka atu hato’o preokupasaun ruma konaba peskiza, favor konatktu:

Salvador Amaral

Email: [Salvador.amaral@menzies.edu.au]

Dr. Lesy Atok

Email: [lesy.atok@menzies.edu.au]

**Autorizasaun Komité étiku:**

Estudu ida ne’e aprovadu ona husi Institutu Nasional de Saúde Públika Timor-Leste, Komité Étiku ba peskiza umana ANU, no Komité Étiku Peskiza Umana husi Eskola Peskiza Saúde Menzies.Karik ita bo’ot iha preokupasaun ka duvida ruma konaba oinsa peskiza ne’e halao, bele kontaktu:

| Gerente Étiku  Komité Étiku Peskiza Umana  Universidade Nasional Australi Telefone: +61261253427 Email: [Human.Ethics.Officer@anu.edu.au](mailto:Human.Ethics.Officer@anu.edu.au) | Departamentu Peskiza Institutu Nasional de Saúde Públika Timor-Leste  Telefone: + +670 7806 2685  Email: uepd insp_tl uepd.insp.tl@gmail.com | Gerente Étiku  Komité Étiku Menzies  Telefone: +61 8 8946 8600, email [NTHREC@menzies.edu.au](mailto:NTHREC@menzies.edu.au) |
| --- | --- | --- |

Obrigado ba konsiderasaun hodi partisipa iha estudu importante ida ne’e. Ita bo’oot nia konhsesimentu sira sai inestimavel atu komprende no mellora ai-han seguru no nutrisaun ih Timor-Leste.

**Written CONSENT FORM for participant**.

Exploring the influence of cultural norms, practices, and community factors on food choices, preparation and food storage in the Timorese community

This Means You Can say NO

- I have read the information sheet, or someone has read it to me clearly in Tetun/English.
- I understand why this study is being done. I know that everything I share will be private and my name will not be used. Only the research team will see my answers.
- I have had the opportunity to ask questions, and I am satisfied with the answers I have received.

NO

YES

- I freely agree to participate in this research project as described and understand that I am free to withdraw at any time during the project without affecting my future interactions with any organizations involved in the study.

YES

NO

Optional consent:

- I agree that the interview will be audio recorded

NO

YES

- I understand that I will be given a signed copy of this document to keep.

YES

NO

Participant Name:…………………………………………………

Signature / Thumb print: ……………………………

Date:…………………………………

Investigator name:………………………………………………

Signatura :………………………………………………………………………

Date:………………………..

FORMULÁRIU KONSENTIMENTU Eskrita ba partisipante.

Esplora influénsia hosi norma kulturál, prátika, no fatór komunitáriu sira kona-ba opsaun ai-han, preparasaun no armazenamentu ai-han iha komunidade Timorense

**Ida-ne'e Signifika katak Ita-boot bele dehan LAE**

- Ha’u lee ona Folla Informasaun Partisipante nian, ka entrevistadór lee ona mai ha’u iha Tetun/Inglés ne’ebé klaru no komprensivu.
- Hau komprende razaun husi peskiza neebe hakerek iha projetu. Hau komprende katak hau nia informasaun sei privatiza no hua nia naran sei la uza. Nune’e mos, ekipa peskiza deit mak bele asesu ba hau niainformasaun sira.
- Ha’u iha ona oportunidade atu husu pergunta, no ha’u satisfeitu ho resposta ne’ebé ha’u hetan.

LAE

SIM

- Ha'u konkorda ho livre atu partisipa iha projetu peskiza ida-ne'e hanesan deskreve ona no komprende katak ha'u livre atu retira iha kualkér tempu durante projetu lahó afeta ha'u nia interasaun sira iha futuru ho organizasaun ruma ne'ebé envolve iha estudu.

SIM

LAE

Konsentimentu opsionál:

- Ha'u konkorda katak entrevista sei grava ho áudiu

LAE

SIM

- Ha'u komprende katak sei fó ba ha'u kópia ida ne'ebé asina hosi dokumentu ida-ne'e atu rai.

LAE

SIM

Partisipante nia naran	:…………………………………………………

Asinatura / Impresaun liman fuan	:……………………………

Data	:…………………………………

Investigadór nia naran	:………………………………………………

Asinatura:………………………………………………………………………

Data	:………………………..
